# A comparative analysis of predictive models of morbidity in intensive care unit after cardiac surgery – Part I: model planning

**DOI:** 10.1186/1472-6947-7-35

**Published:** 2007-11-22

**Authors:** Emanuela Barbini, Gabriele Cevenini, Sabino Scolletta, Bonizella Biagioli, Pierpaolo Giomarelli, Paolo Barbini

**Affiliations:** 1 Department of Physiopathology, Experimental Medicine and Public Health, University of Siena, Siena, Italy; 2 Department of Surgery and Bioengineering, University of Siena, Siena, Italy

## Abstract

**Background:**

Different methods have recently been proposed for predicting morbidity in intensive care units (ICU). The aim of the present study was to critically review a number of approaches for developing models capable of estimating the probability of morbidity in ICU after heart surgery. The study is divided into two parts. In this first part, popular models used to estimate the probability of class membership are grouped into distinct categories according to their underlying mathematical principles. Modelling techniques and intrinsic strengths and weaknesses of each model are analysed and discussed from a theoretical point of view, in consideration of clinical applications.

**Methods:**

Models based on Bayes rule, *k-*nearest neighbour algorithm, logistic regression, scoring systems and artificial neural networks are investigated. Key issues for model design are described. The mathematical treatment of some aspects of model structure is also included for readers interested in developing models, though a full understanding of mathematical relationships is not necessary if the reader is only interested in perceiving the practical meaning of model assumptions, weaknesses and strengths from a user point of view.

**Results:**

Scoring systems are very attractive due to their simplicity of use, although this may undermine their predictive capacity. Logistic regression models are trustworthy tools, although they suffer from the principal limitations of most regression procedures. Bayesian models seem to be a good compromise between complexity and predictive performance, but model recalibration is generally necessary. *k*-nearest neighbour may be a valid non parametric technique, though computational cost and the need for large data storage are major weaknesses of this approach. Artificial neural networks have intrinsic advantages with respect to common statistical models, though the training process may be problematical.

**Conclusion:**

Knowledge of model assumptions and the theoretical strengths and weaknesses of different approaches are fundamental for designing models for estimating the probability of morbidity after heart surgery. However, a rational choice also requires evaluation and comparison of actual performances of locally-developed competitive models in the clinical scenario to obtain satisfactory agreement between local needs and model response. In the second part of this study the above predictive models will therefore be tested on real data acquired in a specialized ICU.

## Background

Different quantitative approaches are frequently used in medical practice to estimate the risk of mortality and morbidity (severity-of-illness) of critical patients [1-4]. Particular attention has been paid to patients after heart surgery in order to improve patient outcomes and improve assessment of the advantages and limits of this costly, high-profile surgery [4]. Many studies have been addressed to developing models to predict the onset of serious postoperative complications [5-8]. However, although heart surgery is one of the commonest operations in the western world and much data is available, it is difficult to model because of the low event rate and the huge number of variables routinely collected. Certain variables are consistently, reliably and reproducibly related to successful prediction of morbidity, whereas many others are not so reliable [7,9-11].

A general point that has given rise to many controversies is the effective exportability of any predictive model to clinical scenarios different from those in which the model was designed [1,5]. Previous papers have demonstrated that morbidity models developed in a specific intensive care unit (ICU) may not be suitable for other ICUs [5,12-14]. However, for benchmarking purposes, one may want to use the same model, say one based on national data, for all hospitals in the country in question. Customizing the model locally to each hospital would make each hospital appear normal, or average, rather than possibly an outlier in relation to the national norm.

Three principal approaches have been used for cardiac surgery risk modelling [4]: logistic regression (LR), Bayes rule and artificial neural networks. The logistic regression approach [15] is perhaps the most common technique used to develop multivariate statistical models to predict morbidity and mortality risk after coronary artery bypass grafting. These models have become very popular in clinical practice since they were transformed into score systems, eliminating computational difficulty in clinical application [7]. In these very attractive models, a risk score is simply estimated by adding several integer coefficients for certain clinical evidence. To this end, any predictive variable has to be categorized by empirically identifying cut-off values which define a risk jump. Although such numerical scoring systems suffer from the subjective choice of cut-off values and a rounding mathematical operation reducing model performance, they have had great success because of their computational simplicity and satisfactory performance in predicting mortality [7,8,11,16-20].

More complex models may be significantly more accurate than simple score models, although they often require special software programs to estimate the risk of morbidity. Algorithms derived from the Bayes theorem can be valid alternatives to score systems [21,22]. Other advanced models, such as artificial neural networks, have also been investigated for improving the accuracy of clinical risk prediction [2,3].

Many studies have compared the performance of different models [23-28]. Asimakopoulos and colleagues [23] investigated the suitability of six different risk stratification systems for estimating mortality risk and comparing surgical performance between institutions or surgeons. They pointed out that all score systems perform moderately at ranking patients and may be useful for patient management. On the contrary, Bridgewater and colleagues [24] tested four score models and demonstrated differences between the British and American heart surgery populations and that North American algorithms are not useful for predicting mortality in the United Kingdom. They therefore advised great care in using methods of this type to compare units and surgeons. Tu and colleagues [25] observed that their LR and artificial neural network models learned similar relationships between patient characteristics and mortality after coronary artery bypass graft surgery. Lippmann and Shahian [26] were not able to demonstrate that artificial neural networks led to significant improvements over logistic or Bayesian models. Knuiman and colleagues [27] compared four approaches for estimating risk of death from coronary heart disease, concluding that there was good, but not excellent, agreement between the methods in estimates of risk for individuals. Marshall and colleagues [28] proved that LR models offer the best overall performance, though other approaches (such as models based on Bayes rule) are good alternatives. More recently, Bayesian statistical models were shown to achieve performances equivalent to those of pure LR but significantly better than LR-derived scoring systems [5].

A salient review of major risk models in cardiac surgery is given in a recent paper [4] that focuses more on fundamental aspects of model development, validation, limitations, current uses and prospects for the future than on model structure. Krumholz [29] and Omar and colleagues [30] highlighted many unresolved issues in modelling risk in cardiovascular medicine and underlined the need to increase literacy regarding risk-adjustment approaches and to pursue methodologically rigorous research to address the gaps in physicians' knowledge of them.

The above considerations prompted us to critically review the characteristics of a number of popular approaches which can be used to estimate the probability of class membership in a unitary framework. Bayes rule, *k-*nearest neighbour method, logistic regression, score systems and artificial neural networks were examined and their performances compared, considering essential issues in model development and evaluating assumptions and adequacy. The present study it is not an exhaustive review of multivariate modelling methods that could be used in the task of predicting the probability of morbidity in heart surgery patients, because only more popular approaches were taken into account.

The study is organized in two distinct parts. In this first part we describe a variety of models to predict morbidity risk probability in the ICU and discuss their theoretical advantages and disadvantages. Model structure, predictive properties and effectiveness of clinical use are analysed and compared, independently of application to real data. The mathematical treatment of some aspects of model structure is also included for readers interested in developing models, though a full understanding of mathematical relationships is not necessary if the reader is only interested in perceiving the practical meaning of model assumptions, weaknesses and strengths from a user point of view. In a second part we will apply the same models to real patient data acquired in a specialized postoperative cardiac ICU and compare their performance, providing guidelines for model choice.

## Key issues for model design

### Clinical framework

Intensive care after heart surgery involves acquisition and analysis of many preoperative, intraoperative and postoperative variables for clinical assessment of patient status. Many may be associated with risk of morbidity after admission to the ICU [7,8].

In the medical literature morbidity has been defined in many different ways but in heart surgery it is always concerned with development of one or more severe cardiovascular, respiratory, neurological, renal, infectious or hemorrhagic complications [7,8,22,31,32].

### Predictive models

Various pattern recognition approaches can be used to design models to separate and classify patients into different prognostic classes [33,34]. Many applications, however, require more than simple classification. In particular, probability estimates are central in medical decision-making, allowing decision makers to incorporate costs/benefits for evaluating alternatives. In the present study eight different predictive models for estimating the probability of morbidity risk on admission to ICU after heart surgery were considered: two Bayesian models [33,35,36], a *k-*nearest neighbour model [33], a logistic regression model [15], two integer score models [5,7] and two feed-forward artificial neural networks (ANNs) [2,34]. Although these models are not the only ones which can be used to estimate the probability of morbidity risk in cardiac surgery patients [27,28], they are certainly the most popular in this field [4], except for nearest-neighbour models which we decided to consider here because of certain interesting theoretical properties which justify their use in other biomedical applications [37,38].

Given the set of chosen predictor variables, ***x***, all models provide a class-conditional probability, *P*(*M *| ***x***), of prognostic risk of morbidity, *M*. Their use for classification implies the choice of a probability threshold value, *P*_*t*_, over/under which patients are recognized to be on a morbid/normal course. In other words, patients are assigned to the morbidity class when *P*(*M *| ***x***) > *P*_*t*_. The choice of *P*_*t*_, depending on the clinical cost of a wrong decision, influences the classification performance of the algorithm [33,39].

Model prediction power is usually expressed by discrimination, calibration and accuracy [1,2,33,40]. Discrimination capacity is a key target to be optimized in any predictive model. An ICU morbidity model shows high discrimination capacity when it correctly distinguishes patients who will develop at least one complication from patients on a normal course. However model generalization properties (namely the ability to show similar performances in different samples from a given population) must be checked when optimizing discrimination capacity during model design [2,34,41]. Great care should be exercised when using models for individual therapeutic choices and prognostic purposes. In this case model optimization to local data (such as customization to the specific institution) may be an important target, because differences between populations may affect model performance [24,29]. This optimization concerns many important procedures that must be followed to ensure the highest possible model discrimination power. Among other things, it includes model choice and updating [42]. When predictive models are used to estimate the probability of morbidity, it is also fundamental to test the extent to which predicted probabilities match observed ones, namely calibration [40,43,44]. Of course, the concept of calibration is meaningless for a hypothetically perfect predictive model. Other important targets are simplicity of model use in clinical routine and effectiveness of implementation.

### Generalization

Generalization is of crucial importance for predictive models designed on a sample data set of correctly classified cases (training data) [34,41]. It is defined as the capacity of the model to maintain the same predictive performance on data not used for training, but belonging to the same population. It is therefore estimated by testing model performance on a different data set of correctly classified cases (testing data). The model generalizes well when predictive errors in testing and training data sets do not differ significantly. Models have to be designed with efficient control of the training process to improve generalization power. Theoretically, the optimal model is the simplest possible model designed on training data which shows the highest possible performance on any other equally representative set of testing data. Excessively complex models tend to overfit, which means they show an error on the training data significantly lower than on the testing data. Overfit is a sort of data storage precluding the learning of prediction rules. It must be avoided since it causes loss of generalization.

To prevent overfitting and improve generalization, various methods can be applied directly during model design [34,41]. The *leave-one-out *method of cross-validation [45] is a suitable approach for all models except ANNs. For ANNs the *early stopping *method is preferred because of computational difficulties, principally due to initialization of the training algorithm. Finally, proper reduction of the number of predictor variables makes model generalization less problematic [39].

#### Leave-one-out

The leave-one-out (LOO) approach is particularly useful in biomedical applications where samples of available cases are generally small, since it allows all cases to be used efficiently for training the model as well as for testing its predictive performance, though this procedure is complex and time consuming. Given *n *correctly classified cases, *n *different combinations of *n *- 1 cases are used for *n *training sessions. The *n *cases left out (one per session) are used to calculate the error. LOO procedure gives a reliable estimate of the prediction error with less bias than other cross-validation methods and it allows good control of model generalization capacity using all available data for training. In most cases, however, it may be better also to evaluate model generalization on a test set of data not used in the training process.

#### Early stopping

The early stopping (ES) method divides the available data into training and validation sets. The control of generalization for ANNs is carried out directly during an iterative process of learning. At each iteration (epoch) the ANN is trained using training data, and the error is computed on both the training and validation sets. During the first phase of learning, both training and validation errors decrease, but, from a given epoch (the ES epoch) onward, the validation error increases as the network starts to overfit, while the training error continues to decrease. Overfitting can be prevented by stopping the training process at the ES epoch [34].

The ES method is largely employed for training ANNs because of its very fast computational time. However it is crucial to realize that the validation error may not be a good estimate of the generalization error. One method for obtaining an unbiased estimate of the generalization error is to test ANN on a third set of data that was not used during the training process. When comparing different models using the same training and testing data sets, it can however be convenient to avoid the use of three data sets.

#### Stepwise feature selection

Another well-known source of generalization loss is the use of too many predictor variables in the model [33,34,39,41,46]: the greater the number of predictor variables, the greater the number of model parameters to be estimated. So, for a given training set, model parameter estimates sharply deteriorate as the number of predictor variables increases. The immediate consequences are a significant loss of model generalization.

Different groups of predictor variables may provide largely overlapping information and subsets of them may maintain similar predictive power. It is therefore convenient to select the best minimum subset of predictor variables (also named features). To select a minimum subset of features allowing optimization of model generalization, a computer-aided stepwise technique can be used [47], together with the LOO (or ES for ANNs) method described above. At each step of the process, a variable is entered or removed from the predictor subset on the basis of its contribution to a statistically significant decrease in the LOO discrimination error (or ES error for ANNs). The stepwise process stops when no variable satisfies the criterion for inclusion or removal.

### Discrimination: receiver operating characteristic curves

The discrimination capacity of an ICU morbidity predictive model assesses model performance in correctly assigning patients to classes with different outcomes. Many criteria exist for evaluating discrimination capacity [33,34]. A common way for binary diagnostic models is to evaluate sensitivity (SE) and specificity (SP), describing the fractions of correctly classified morbid and normal patients, respectively [48]. Generally, SE and SP depend on the chosen probability threshold (decision probability, *P*_*t*_) to which the model-predicted probability of morbidity is compared [48].

**Figure 1:**
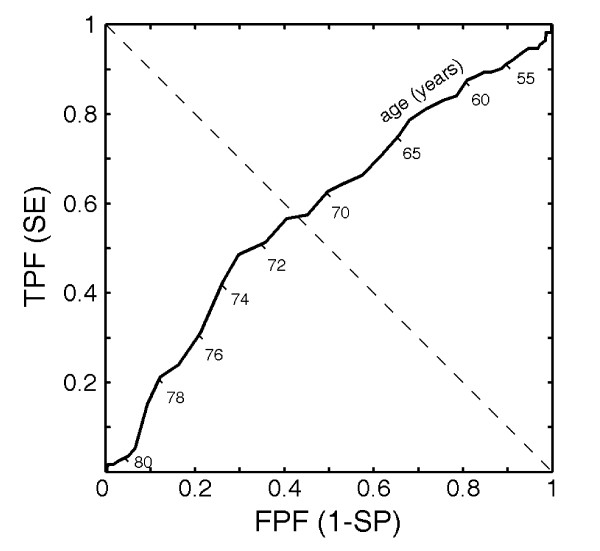
Example of a receiver operating characteristic (ROC) curve obtained using patient age as only predictor. Numerical values of ages are plotted under the ROC line. FPF and TPF denote false-positive and true-positive fractions, respectively. Diagonal dashed line intersects ROC curve at point where sensitivity (SE) and specificity (SP) are equal (71 years in the example).

A receiver operating characteristic (ROC) curve is a graphic representation of the relationship between the true-positive fraction (TPF = SE) and false-positive fraction (FPF = 1 - SP) obtained for all possible choices of *P*_*t*_. Figure 1 illustrates an example of an empirical ROC curve, obtained from sample data in an ICU, using patient age as the only predictor, the numerical values of which are plotted under the ROC line. Note that the intersection between the dashed diagonal and ROC lines indicates the point where SE equals SP. The choice of a decision probability threshold *P*_*t *_corresponding to the ROC curve point of equal SE and SP leads to the same error rate in identifying normal and morbid patients. Different discrimination criteria, i.e. different pairs of SE and SP, may be chosen, depending on the clinical cost of a wrong decision [33].

In medical applications the area under the ROC curve (AUC) is the most commonly used global index of discrimination capacity, although alternative indices can be used [49-51]. The AUC can be interpreted as the probability that a patient randomly selected from the morbidity group will have a diagnostic marker indicating greater risk than a randomly selected patient from the normal course group [52].

AUC can be readily calculated from data samples using an empirical method involving numerical integration of the ROC curve [51]. The bootstrap resampling method can be used to estimate confidence intervals for AUC [51,53]. Bootstrapping amounts to resampling the original data set (parent sample), with replacement, to generate a number of bootstrap samples. In traditional applications the size of a bootstrap sample equals the parent sample size.

### Calibration

The agreement between model-predicted and true probabilities is known as calibration or goodness of fit [2,15,40]. It is independent of discrimination, since there are models that have good discrimination but poor calibration. A well-calibrated model gives probability values that can be reliably associated with the true risk of developing outcomes.

For dichotomous outcomes, true risk probabilities cannot intrinsically be known. In fact, retrospective data only provides dichotomous responses, such as presence or absence of morbidity. Nevertheless, it is sometimes useful to estimate the occurrence of these events using a continuous scale. For example, in ICU morbidity prediction, a probabilistic estimate of the patient's outcome is usually preferred to a simpler binary decision rule.

When evaluating the calibration of LR models with dichotomous outcomes, the Hosmer-Lemeshow (HL) method is a commonly used goodness-of-fit test, which is based on chi-squared statistics comparing expected and observed frequencies of outcomes [15]. Two formulations of HL statistics exist, depending on whether the statistics are derived using fixed deciles of risk, Ĥ-statistics, or by partitioning observations into equal sized groups according to their predicted probabilities, Ĉ-statistics. Generally, Ĉ-statistics is preferred because it avoids empty groups, despite being heavily dependent on sample size and grouping criterion [1].

Although the HL test was developed for LR models and its Ĉ-statistics may not be completely appropriate for models with discrete outputs (such as score systems), it can nevertheless be applied to any predictive model, sometimes opportunely recalibrated to improve goodness of fit.

### Accuracy

The mean squared error, MSE, between model predicted probability and observed binary outcomes is often taken as a single global index of model prediction performance and is known as accuracy. For a sample of *n *cases, it is defined as

(1) $$MSE=\sum_{i=1}^{n}\frac{{\left[O_{i}-P(M|x_{i})\right]}^{2}}{n}$$

where *O*_*i *_is the observed outcome in the i-th case, which takes a value of 1 or 0 for morbid and normal patients, respectively, and ***x***_*i *_is the corresponding predictor vector.

MSE can be decomposed into three addends, that account separately for calibration, discrimination and sample data properties [54].

### Model recalibration

A major requirement of any predictive model is that it adequately reflect risk in the population evaluated. When model performance is inadequate, the user can apply suitable methods to improve model function [42-44,55,56]. Some methods change model discrimination and calibration, whereas others only modify model calibration [43]. In other words, in the case of a predictive model of morbidity probability with good discrimination and poor calibration, it is possible to improve calibration without modifying discrimination capacity [57]. In fact it is intuitive that a monotonic mathematical transformation of model predicted probabilities does not change AUC but may change calibration.

De Long and colleagues [43] applied two simple monotonic transformations to the mortality probability estimated by logistic regression in patients after coronary artery bypass surgery. The first transformation (prevalence correction) is a constant correction term which was added to each patient's risk score; the second (modelling the risk score) recalibrates the model predicted probability by a linear regression approach.

Complex models designed to optimize performance in discriminating binary events tend to create a bimodal probability distribution. In this case the relationship between actual and model predicted probabilities could be better approximated by using other appropriate methods (e.g. polynomial regression, B-splines or similar). For example a convenient recalibration of model predicted probability may be obtained by cubic monotonic polynomial fitting:

(2) $$P_{rec}=\frac{1}{1+\exp(a+bP+cP^{2}+dP^{3})}$$

where *d *must be set equal to *c*^2^/(3*b*) to ensure that the cubic polynomial is a monotonic function. *P *concisely indicates the morbidity risk probability, that is *P*(*M *| ***x***), and *P*_*rec *_is the corresponding recalibrated value. The relationship in equation2 ensures that the recalibrated probability takes values between 0 and 1.

Polynomial coefficients *a*, *b *and *c *can be estimated from training data using a least square algorithm to minimize the expression of MSE. A minimum MSE means a maximum calibration, because the monotonic transformation of equation2 does not influence model discrimination or sample properties, that is, the other two addends of the linear decomposition of MSE (see previous subsection). Recalibration effectiveness can also be evaluated on testing data.

Although different more complex recalibration functions may provide better recalibration on training data, they are inadvisable because they can cause loss of generalization. Recalibration should only be done for models for which the benefit is evident. In particular, well-designed LR models are usually also well calibrated.

### Clinical implementation

In addition to predictive capacity, other key characteristics for the clinical success of a predictive model, are simplicity and effectiveness of application, which include easy customization to local conditions and/or institutions, easy updating with new data, computational facility, tolerance to missing data and ability to provide supplementary clinical information.

#### Customization

Risk prediction models developed in a different institution require local validation and tuning before they can be used to provide risk-adjusted outcomes [5]. Model customization to local conditions is necessary because clinical practices are difficult to standardize and because patient populations differ. For example, different medical protocols could provide different sets and types of variables at different times of measurement. Model customization implies that feature selection be repeated and new model parameters be estimated from locally-available data. A periodic reassessment to account for new measurements and protocols and to ensure that tuning is maintained, is also beneficial.

#### Updating

The capacity of a predictive model to learn from new correctly-classified cases, day by day, is an import index of quality, especially in clinical practice where data is usually scarce and training on new data becomes of crucial importance. For any model, the whole model design should theoretically be repeated when adding a new case to a training set. However, only simple and rapid procedures of model updating are generally acceptable, because the largest proportion of care and time should be dedicated to optimization of patient treatment.

#### Computational facility

Score models are much preferred by clinicians because they do not require the use of computers for classification of a new test case. Computation facility, greatly appreciated in clinical practice, should however be carefully evaluated in conjunction with predictive accuracy, because the two are often inversely correlated. Too many simple models may be useless or even counterproductive, giving a misleading estimation of a patient's clinical risk.

Furthermore, the software for data acquisition, management and processing may also be model dependent: for example, models requiring fewer predictive variables have a simpler data entry procedure.

#### Tolerance to missing data

In clinical practice, data can be missing for many reasons, such as the impossibility of making a reading or priority of prompt clinical intervention over data acquisition. In certain types of models, missing data can be replaced by suitable procedures, with negligible or tolerable loss of predictive performance.

#### Supplementary clinical information

Additional information of clinical interest can also be obtained. For example, some models allow newly classified clinical cases to be simply associated with previous similar cases, providing a useful tool for interpretative and comparative diagnosis. Other models are particularly convenient for simulation purposes, such as for evaluating changes in prognostic probability due, for example, to contemplated therapy which may modify the values of certain predictive variables.

## Model description

### Bayesian models

Let ***x ***be an observed predictor vector and let our aim be to determine whether ***x ***belongs to the class of morbidity class or of normal course. The Bayes rule enables prediction of the posterior conditional probability of morbidity class, as follows [33,35,36,39,58]

(3) $$P(M|x)=\frac{p(x|M)P(M)}{p(x|M)P(M)+p(x|N)P(N)}$$

where *P*(*M*) is the prior probability of morbidity, *P*(*N*) = 1 - *P*(*M*) is the prior probability of normal course and *p*(***x ***| *M*), and *p*(***x ***| *N*) are the class-conditional probability density functions (CPDFs) for morbid and normally recovering patients, respectively.

Likewise, the posterior conditional probability of normal course is

(4) $$P(N|x)=1-P(M|x)=\frac{p(x|N)P(N)}{p(x|M)P(M)+p(x|N)P(N)}$$

A reasonable discrimination criterion would be to assign patient ***x ***to the class with the largest posterior probability, thus obtaining the Bayes decision rule for minimum error [33]. This means setting the posterior class-conditional probability threshold (*P*_*t*_) equal to 0.5, that is, ***x ***is assigned to class *M *if *P*(*M *| ***x***) is greater than 0.5 and otherwise to class *N*. However, the decision rule may be formulated using somewhat different reasoning. Often, in medical applications, the decision rule must account for the cost of a wrong decision. In this case, a cost can be assigned to each correct and wrong decision and *P*_*t *_is chosen to obtain the minimum risk decision rule [33]. From a purely mathematical point of view, the selection of costs is equivalent to a change in prior probabilities. Consequently, the decision rule only gives the minimum risk if *P*(*M*) and *P*(*N*) do not change, but if prior probabilities vary, the fixed threshold value no longer gives the minimum achievable risk.

When prior probabilities may vary, it is possible to design the threshold value so that the maximum possible risk is minimized, regardless of changes in prior probabilities (minimax risk decision rule). In particular, setting the cost of a correct decision equal to 0 and assuming equal costs for wrong decisions, the boundary for the minimax risk decision rule satisfies the following relationship [33]

(5) $$\int_{\Gamma_{N}}p(x|M)dx=\int_{\Gamma_{M}}p(x|N)dx$$

where Γ_*N *_and Γ_*M *_are the regions in the ***x ***domain where we decide that ***x ***belongs to classes *N *and *M*, respectively. In other words, the decision boundary is selected such that the error percentages for both classes are equal. This amounts to assigning equal values to SE and SP.

Besides prior probabilities, the above Bayes rule requires that the class-conditional probability density functions be known for morbid and normally recovering patients. In the clinical field, such CPDFs must be estimated using a finite number of observed cases. If no assumptions are made, these functions have to be estimated from the training set using non-parametric methods [39]. However, the great majority of applications still rely on various parametric hypotheses. Very often normal CPDFs are assumed, since in many cases this choice provides a simple and robust method of discrimination, especially when many variables are available and a subset of them has to be selected [5,33,39,59]. Under this hypothesis the CPDF of group *i *(*i *= *M *or *N*) is given by the multivariate normal probability density

(6) $$p(x|i)=\frac{1}{{\left(2\pi \right)}^{d/2}}|\Sigma_{i}{|}^{-1/2}\exp\left\{-\frac{1}{2}{\left(x-\mu_{i}\right)}^{⊤}\Sigma_{i}^{-1}(x-\mu_{i})\right\}$$

where ***μ***_*i *_and **Σ**_*i *_are the mean and the covariance matrix of class *i*, *d *the number of predictor variables used for discrimination and superscript T indicates matrix transposition. The CPDFs in equation6 can be easily estimated and locally tuned, since they require only the calculation of group means (***μ***_*M *_and ***μ***_*N*_) and covariance matrices (**Σ**_*M *_and **Σ**_*N*_) from training data.

Based on hypotheses about class covariance matrices, two parametric Bayesian models can be developed [33]: the first approach estimates a different covariance matrix for each class from the training set and leads to the so-called Bayes quadratic (BQ) classifier, because the decision boundary is given by a quadratic form in ***x***, while the second approach assumes homoscedastic distributions and leads to the Bayes linear (BL) classifier characterized by a linear decision boundary. For the BL model the covariance matrix is estimated as the pooled within-sample covariance matrix.

### The *k*-nearest neighbour model

The *k*-nearest neighbour (*k*NN) allocation rule is a method for classifying patients based on non parametric estimation of class-conditional probability density functions [33]. Briefly, the training phase of the algorithm consists of storing the predictor vectors and class labels of the training samples and mapping the class labels of training cases into multidimensional predictor space. In the classification phase, the same predictors as before are acquired for the test case (the class of which is unknown). Distances from the new vector to all stored vectors are computed and *k *closest cases are selected. The new case is predicted to belong to the most numerous class in the neighbourhood of *k *closest cases. Euclidean distance is usually used. Thus multidimensional predictor space can be simply partitioned into regions by assigning each point in the space to the class which is the most popular among the *k *nearest training cases.

Neighbourhood size is essential in building the *k*NN classifier because it can strongly influence the quality of predictions; larger values of *k *can reduce the effect of noise on the classification but make boundaries between classes less distinct. Typically, *k *should depend on the size of the training set [33]. A common and simple heuristic choice is setting *k *equal to the square root of the number (*n*) of cases in the training set [60], although different choices are possible (for more details see Discussion). In Part II of our study the value of *k *was set at

(7) $$k=\text{round}\left(\sqrt{n}\right)$$

where the operator round indicates rounding to the nearest integer.

The model predicted conditional probability of morbidity can be estimated as follows

(8) *P*(*M *| ***x***) = *k*_*M*_/*k*

where *k*_*M *_is the number of morbid cases in the training neighbourhood of *k *cases. In particular, equation8 corresponds to the Bayes decision rule for minimum error if the prior probability of morbidity *P*(*M*) is assumed equal to *n*_*M*_/*n*, where *n*_*M *_indicates the number of morbid cases in the training set [33].

### Logistic regression model

Binomial (or binary) logistic regression is a form of regression which can be used to predict outcome probability when the dependent variable is dichotomous and the independent predictor variables are of any type [15,33,39]. Predictors may be a combination of continuous and categorical variables. Multinomial logistic regression exists to handle the case of dependent variables with more than two classes [61,62]. When multiple classes of the dependent variable can be ranked, then ordinal logistic regression is preferred to multinomial logistic regression [61]. Continuous variables are not used as dependent variables in logistic regression.

When considering morbidity of ICU patients the outcome is a dichotomous variable which can be binarized as 1 and 0 for morbidity and normal clinical course, respectively (binary LR). Though the binary LR model is used for a categorical dichotomous variable, the output is a continuous function (S-shaped curve) that represents the outcome probabilities associated with being in a specific category of the dependent variable. The conditional probabilities of outcome are expressed as

(9) $$\begin{array}{l} P(M|x)=\frac{\exp(\alpha +\beta^{⊤}x)}{1+\exp(\alpha +\beta^{⊤}x)}\\ P(N|x)=1-P(M|x)=\frac{1}{1+\exp(\alpha +\beta^{⊤}x)} \end{array}$$

where the constant *α *and the vector of coefficients *β *are the model parameters. Data from a training set are used to obtain the maximum likelihood estimates of these LR model parameters [48].

The ratio *P*(*M *| ***x***)/[1 - *P*(*M *| ***x***)], termed the odds of morbidity, is given by

(10) $$\frac{P(M|x)}{1-P(M|x)}=\exp(\alpha +\beta^{⊤}x)$$

so that the natural logarithm of the odds (called the logit)

(11) $$\ln\left[\frac{P(M|x)}{1-P(M|x)}\right]=\alpha +\beta^{⊤}x$$

is a linear function of ***x***. The parameters of the logistic regression model can therefore be interpreted as the regression coefficients of equation11 and exponentiating these parameters provides the odds ratio corresponding to a one unit change in each independent predictor variable.

### Integer score models

#### The Higgins score model

Higgins and colleagues proposed a procedure for designing a simple-to-use score model (HS model) for predicting morbidity risk on admission to ICU after coronary artery bypass grafting [7]. The design of this type of model first requires the development of a LR model as discussed in the previous subsection. After the LR model has been built using a forward stepwise selection procedure for the choice of a subset of predictor variables *x*_*i *_(*i *= 1, 2,...,*d*), each continuous predictor is categorized using a locally weighted scatterplot procedure to subjectively identify cut-off points on the basis of training data. Then a new LR model is again developed using predictors as categorical variables. Finally, a numeric score *s*_*i *_is given to each predictor by multiplying the estimate of the corresponding parameter *β*_*i *_of this second LR model by 2 and rounding the result to the nearest integer [7]. Given a test patient ***x ***to classify, all observed predictor values (*x*_*i*_) are compared to the associated model cut-off points. Whenever this comparison gives a categorical variable value corresponding to an increased risk of morbidity, the associated score *s*_*i *_is added to model score. Thus the model score for the test patient ***x ***is obtained as follows

(12) $$s=\sum_{i=1}^{d}\lambda_{i}s_{i}$$

where *d *is the number of predictors in the model, *s*_*i *_the score associated with the *i*^th ^predictor and *λ*_*i *_a coefficient assigned a value of 0 or 1 after comparison of *x*_*i *_with the corresponding cut-off point.

In the original paper of Higgins and colleagues the risk levels of test patients were categorized on the basis of similar outcomes in the training set, because mortality and morbidity risks were both taken into account in developing the score model. For example, in that paper patients with a score *s *less than 5 at ICU admission were classified at a risk level of less than 1% for mortality and less than 5% for morbidity [7]. When the model is developed accounting only for one type of outcome (morbidity), an alternative approach can be to directly estimate the probability of morbidity for a test patient ***x ***by dividing the value obtained for *s *by its maximum possible value given by

(13) $$s_{\max}=\sum_{i=1}^{d}s_{i}$$

#### Direct score model

An alternative approach to the Higgins one can be to directly select a weighted combination of binarized predictor variables to be summed to obtain the model output as an integer score of morbidity risk. We refer to this model as the direct score (DS) model. More in detail, in this approach all predictor variables have to be coded to binary values (0 or 1) on the basis of their association with ICU morbidity. Thus continuous variables are binarized by selecting suitable cut-off points, defining corresponding values for sensitivity and specificity [48]. Once a cut-off point for a continuous variable has been chosen, the resulting 2 × 2 classification matrix on training data allows the computation of SE = TP/(TP+FN) and SP = TN/(TN+FP), where TP, TN, FP and FN are true positives, true negatives, false positives and false negatives, respectively [63]. Of course, SE and SP both vary, changing the cut-off point. A suitable choice can be made setting the cut-off point so that SE and SP are equal; with reference to the example of Figure 1, this choice corresponds to an age of 71 years. Thus each continuous variable can be binarized, comparing its value with the established cut-off point.

Whenever a variable is binary coded, its discrimination power must be evaluated on the basis of the corresponding confidence interval of the odds ratio [64], so that only binary variables with an odds ratio significantly greater than 1 are considered likely to be chosen as risk predictors from the final selection of model features carried out using a forward stepwise method.

At the first step, the forward method of feature selection chooses the binary variable with the highest AUC on the training data. At any subsequent step the variable giving the highest increment to AUC is entered. The procedure stops when no appreciable increment to AUC occurs. The model integer score is simply computed by summing the binary values of the selected variables. At each step, formerly selected variables are also reconsidered for entry into the model. This allows the model to give different weights (scores) to each predictor variable by adding its corresponding binary value several times. Like the HS model, the output of the DS system for test patient ***x ***can therefore be obtained using equation12, where *s*_*i *_is the weight given to the *i*^th ^model predictor. Since the DS model does not have a parametric mathematical structure, backward sessions and the LOO procedure for cross-validation cannot be applied.

The class-conditional model probability of morbidity risk, *P*(*M *| ***x***), is estimated dividing the integer score obtained for patient ***x ***by its maximum possible value, given again by equation13.

### Artificial neural networks

The starting point of artificial neural networks is quite different from that of statistical models. Neural networks are generic learning systems [34]. Although ANNs directly estimate class-conditional probabilities, the model design parameters are meaningless [2,34]. The LR approach also estimates such probabilities directly but the model parameters have simple interpretations in terms of natural logarithms of odds, that are easily understood by subject-matter researchers.

Two feed-forward ANN architectures are considered here:

• ANN1: the simplest possible feed-forward ANN architecture, with only one output neuron, the so-called single layer perceptron;

• ANN2: containing two layers, a hidden layer with two neurons and an output layer with one neuron, respectively.

The number of inputs of ANN1 and ANN2 is equal to the dimension *d *of the vector ***x ***of selected features. Both have one output neuron designed to estimate the probability of morbidity risk through a logistic sigmoid (*logsig*) activation function, generating an output *y *between 0 and 1. The function *logsig *is defined as

(14) $$y=\frac{1}{1+\exp(-g)}$$

where *g *is

(15) *g *= ***w***^⊤^***u ***+ *b*

and ***u ***is the neuron input vector, ***w ***the weight vector and *b *the bias [34]. It is interesting to observe that equations14 and 15 express the same mathematical relationship as equation 9.

For the single layer network ANN1, the input vector ***u ***of the output *logsig *neuron is simply the feature vector ***x ***of the test patient, whereas for ANN2 it is the outputs *y*_*i *_(*i *= 1,2) of the hidden layer characterized by the following hyperbolic tangent sigmoidal (*tansig*) activation function

(16) $$y_{i}=\tanh(g_{i})=\frac{\exp(g_{i})-\exp(-g_{i})}{\exp(g_{i})+\exp(-g_{i})}=\frac{2}{1+\exp(-2g_{i})}-1$$

where *g*_*i *_is defined as for equation15.

Each predictor variable *x*_*i *_(*i *= 1, 2,...,*d*) is standardized before presentation to the network, so as to have zero mean and unit standard deviation, because standardization has been shown to increase the efficiency of ANN training. Weight vectors and biases are the ANN parameters to estimate by iterative learning procedures. Of course, ANN2 has more parameters to estimate than ANN1.

Feed-forward ANNs for classification, designed with one output *logsig *activation function, have proven able to provide reliable estimates of class-conditional probabilities, such as *P*(*M *| ***x***) and *P*(*N *| ***x***) [34].

The ANN output (target) is set equal to 1 for training examples of morbid and 0 for normal course patients. Usually the mean square error MSE, i.e. the mean of the squared differences between real and network predicted outputs, is minimized to estimate ANN parameters. The dependence of the solution on training procedure initialization can be limited by running many training sessions from as many different randomly-selected initial conditions, and choosing the session corresponding to the 50^th ^sorted value (median) of AUC.

## Discussion

In any application, the choice of an optimal model is rarely univocal and cannot be made a priori. For clinical decisions, users should prefer simple intuitive models to complex ones, but this preference should be evaluated in the light of model fit to the experimental data. An excessively simple model may be unable to provide good fit of the data. On the other hand, the prediction results obtained using highly complex models, which may fit the data very well, may not allow an immediate and intuitive interpretation in terms of cause and effect from a clinical point of view. The choice of a predictive model can therefore generally be made only a posteriori, after the training process and after model performance and its characteristics have been evaluated on a suitable test set. In Part II of this study, the working of the above models will be compared using clinical data acquired in a specialized ICU.

In the following subsections the key peculiarities of each predictive model are analysed from a purely theoretical point of view, independent of clinical scenarios and real data. Model advantages and disadvantages are evaluated and discussed on the basis of design procedures and speculative characteristics.

### Bayesian models

Assuming that class prior probabilities do not vary, knowledge of the prior probability and class-conditional probability density function for each class allows the Bayes decision rule to be optimal in the sense that it minimizes the probability of error or the expected cost [5,33]. However, even if the prior probabilities vary, the minimax test can be used to design a Bayes classifier to minimize maximum possible risk [33].

The BL model originates from the assumption of normal CPDF for each class with equal covariance matrices. Nevertheless, in many cases, the simplicity and robustness of the BL model compensate the loss of performance occasioned by non-normality or non-homoscedasticity [33,39,59]. This model is easy to implement in clinical decision-making, requiring only the knowledge of the different class mean vectors and only one covariance matrix which can be estimated by a suitable training set [39]. Its simplicity of application in clinical practice is another significant advantage of this approach with respect to other methods. In fact for recognizing morbidity, the Bayes decision rule can be expressed as a linear function of the observation vector, and computed with a hand calculator [33].

The BQ model is generally more robust than BL though its clinical application may be slightly more complex and time consuming from a computational point of view, because the decision rule is expressed as a quadratic function of the observation vector.

The BL and BQ models can be easily tailored to a given institution, because their local customization only requires the estimation of class mean vectors and covariance matrices (only one covariance matrix for BL). Furthermore both models can be updated in a straightforward way by entering each new correctly classified case in the training set, since this simply involves updating mean vector and covariance matrix estimates by the following recursive relationships

(17) $$\{\begin{array}{c} \hat{\mu }(n+1)=(1-\delta )\hat{\mu }(n)+\delta x(n+1)\\ \hat{\Sigma }(n+1)=(1-\delta )\left\{\hat{\Sigma }(n)+\delta \left[x(n+1)-\hat{\mu }(n)\right]{\left[x(n+1)-\hat{\mu }(n)\right]}^{⊤}\right\} \end{array}$$

where $\hat{\mu }$(*n *+ 1) and $\hat{\Sigma }$(*n *+ 1) are the estimates of mean vector and covariance matrix updated according to the new case ***x***(*n *+ 1) and the previous estimates $\hat{\mu }$(*n*) and $\hat{\Sigma }$(*n*). The parameter *δ *is a coefficient weighting later observations. If *δ *is set equal to 1/(*n *+ 1) all observations have the same weight irrespective of the time of occurrence.

Poor calibration is generally a weakness of Bayes models. It may therefore be convenient to perform a recalibration to improve the model's ability to estimate the correct probability of morbid or normal outcomes in ICU patients.

### The *k*-nearest neighbour model

The *k*NN algorithm uses training data directly for classification. A key advantage of this non-parametric approach is that it does not make any statistical assumption about the data, thus enabling an arbitrary decision boundary. *k*NN models are also very easy to update with new data: each new correctly classified patient can be added to the training database and used to classify subsequent cases.

Another strength of *k*NN algorithms over other approaches is that any new test case can be analysed and interpreted by comparing it with its *k *neighbours. This provides useful insights for clinical interpretation of the classification results, helping in comparative diagnosis.

However, it is clear that the choice of *k *is critical, because it represents a trade-off between local and global approximations of the model. The optimal neighbourhood size depends on training data, that is, on the number of cases and features in the training set. According to the asymptotic reasoning of Fukunaga [33], *k *should be chosen so as to be proportional to *n*^4/(*d*+4)^, where *n *is the number of training cases and *d *is the dimension of the vector for which the nearest-neighbour density has to be estimated, with the proportionality constant depending on the underlying density [65,66]. Nevertheless, in practice, the choice of *k *is often necessarily based on the square root of all possible candidates, that is $k=\sqrt{n}$[60]. Alternatively, one can also use objective criteria such as generalized cross validation, which involves determining classification accuracy for multiple partitions of the input cases used in training [65].

The choice of metric for calculating distances between cases can be another critical point in the design of *k*NN models, because model performance also depends on the measure of distance used. In Part II of the present study, where the classical Euclidean distance was used, each predictor variable was standardized by subtracting its mean value and dividing by its standard deviation.

Computational cost and the need for large data storage are weaknesses of this algorithm. In fact the *k*NN algorithm searches through all the dataset looking for the most similar instances. Although fast computers are available today, this is a time consuming process and may be critical in data mining where very large databases are analyzed. Of course, training and customization to a local institution only become valid when many cases have been recorded. For a large database, the distance between the new test case and each training cases must be calculated, and then distances must be ranked. However, the computational complexity of the *k*NN model can be reduced in several ways [60], for example, sample selection, storing only a reduced sample (such as prototypical examples of each class) of the historic database so that fewer distances have to be computed, or box generation, pre-processing the whole training set using a balanced box decomposition tree [67]. On the other hand, techniques for reduction of computational complexity generally make updating the model more complex.

Finally, *k*NN is also sensitive to the presence of variables that are irrelevant for classification purposes. All non-parametric techniques have a tendency to overfit the model when the number of variables used is too large.

### Logistic regression model

Logistic regression allows one to predict a discrete outcome, such as group membership, from a set of independent predictor variables that may be continuous, discrete, dichotomous, or a mix of any of these. Indeed, LR does not require that the independent variables be interval and unbounded.

Since the natural logarithm of the odds is a linear function of the observed variables (see equation11), the application of a binary LR decision rule may seem to have affinity with the BL model [39]. However, a key advantage of binary LR is that only *d *+ 1 parameters (constant *α *and vector *β*), where *d *indicates the number of independent predictor variables in the model, are estimated from the training set, whereas assuming normal CPDF for each class with equal covariance matrices, the BL model requires estimation of many more parameters (mean vectors of the two classes and pooled within-sample covariance matrix), that is (*d*^2 ^+ 5*d*)/2. Moreover the natural logarithm of the odds is linear in ***x ***for a range of different assumptions about the CPDF of the independent variables, so that the logistic model is optimal under a wide range of data types and the assumption of logistic form of the posterior probabilities generally yields a reasonable decision rule [39].

The performance of LR models has often been compared to that of BL classifiers in discrimination problems. A common conclusion is that the LR approach is preferable to BL when CPDFs are clearly non-normal or covariance matrices are manifestly different. Otherwise, the two approaches generally give very similar results [33,39].

It should also be noted that logistic regression does not assume a linear relationship between the dependent and independent variables. Indeed, by assuming a linear relationship between the natural logarithm of the odds and the predictor variables, it may handle a variety of nonlinear effects.

Despite the above lack of constraints on the type of independent variables and their distribution, LR should only be used if certain assumptions about these variables are true [15]. As in most regression procedures, LR is very sensitive to large linear correlations between the predictor variables in the model [48]. Another crucial point is the assumption of inclusion of all relevant variables and exclusion of all irrelevant ones in the regression model. Furthermore all effects are additive; LR does not account for interaction effects except when interaction terms (usually products of standardized independents) are created as additional variables in the analysis.

A key assumption in LR models is that error terms are independent. Violations of this assumption can have serious effects. This occurs, for example, in correlated samples and repeated measure designs, such as matched-pair studies and time-series data. Variations of logistic regression are available to fit correlated or clustered observations [15,68-70].

A main weakness of LR is that outliers can affect the results significantly. The researcher should analyse standardized residuals for outliers and consider removing them or modelling them separately.

A final remark is that the LR model is not simple to update with new training data although periodic full retraining may not cause excessive problems. Like Bayesian models, its clinical implementation may require the use of a personal computer, which also allows much other medical information to be obtained in the ICU.

### Integer score models

#### The Higgins score model

The Higgins score model is derived from a LR model using a procedure which transforms independent continuous variables into categorical variables and LR coefficients into integer scores. These transformations make the HS model very attractive for clinical applications, because its routine application in the ICU is simple and does not require the use of a personal computer. However, this implies a loss of performance with respect to the original LR model [5]. The training process is complicated and partly subjective, the model is less flexible and lower predictive accuracy can be expected, model customization is difficult, updating is practically impossible without retraining and no other reliable clinical information can be obtained apart from discrimination. All scoring systems provide a discrete estimate of the class-conditional probability of morbidity risk and consequently poor calibration.

#### Direct score model

The direct score system is the simplest model analysed in the present study. Like the Higgins score model, it suffers from the empirical nature of the process used for dichotomizing continuous variables, which requires the choice of suitable cut-off points. However setting the cut-off points so as to obtain equal values for SE and SP makes this choice less subjective and allows the same procedure to be repeated in different ICUs.

The clinical use of this model is very simple, because it only requires comparison of predictor values measured in the test patient with the corresponding cut-off points and then summing of integers to obtain the model-predicted morbidity score. This great simplicity may not enable sufficient predictive accuracy. Moreover the use of this type of model implies a discrete estimate of class-conditional probability and subsequently poor calibration. Finally, updating requires complete retraining.

### Artificial neural networks

Artificial neural networks have recently had many successful applications in medicine [1-4,45]. Key advantages with respect to common statistical models are: no statistical assumption about data distribution is required; no mathematical model has to be defined; not too much complex network architecture has to be designed to suitably approximate any unknown nonlinear relationships between predictor input variables and output probabilities; the distributed structure of the network may account for correlations among input variables; ANNs can be trained with examples like human brains to use acquired knowledge in decision making; the training process can be controlled to avoid overfitting and loss of generalization capacity.

To solve complex problems many attempts can be made, increasing the complexity of the architecture by trial and error or using sophisticated techniques, such as growing, pruning and genetic algorithms, to find optimal ANN structure [34]. However prediction of morbidity in the ICU generally does not require excessively complex modelling, at the risk of uncontrollable overfitting. In this case it may be preferable to favour system generalization by using simple models that approximate the real phenomenon by means of few design parameters.

Considerable difficulties may arise when designing and using ANNs. The training process is difficult and not univocal: the problem of initialization is all but trivial; as in all nonlinear procedures, many different solutions, which are difficult to compare and interpret, may be obtained; the complexity of ANN architecture is only roughly definable in terms of number of neurons, layers and connections.

For the above reasons, only two simple ANN architectures are considered in Part II of the present study. ANN1 is the simplest possible feed-forward ANN architecture, having only one output neuron, the so-called single layer perceptron. ANN2 has two layers, a hidden layer with two neurons and an output layer with one neuron. When predicting the risk of morbidity in the ICU these two architectures allow a good compromise between ANN capacity in describing training data and generalization power. In particular, the simpler one (ANN1) gives the same input-output nonlinear mathematical relationship as the LR model, though its parameters are estimated by a different computational procedure.

The high flexibility and sophisticated training procedures of ANN models allow very good customization to data of local institutions, but continuous updating is practically impossible. Periodic retraining is inadvisable, because of the complexity of the training process. ANNs should therefore be trained once and for all, using a sufficiently large number of cases representative of the study population.

## Conclusion

Different approaches for developing predictive models of morbidity in cardiac postoperative intensive care units have been reviewed in a unitary framework from a theoretical point of view. We grouped popular methods into distinct categories according to their underlying mathematical principles. Modelling techniques and intrinsic advantages and disadvantages of each predictive model have been discussed with a view to clinical applications. Main strengths and weaknesses are summarized in Table 1. Briefly:

**Table 1:** Main strengths and weaknesses of popular predictive models.

**Model**	**Strengths**	**Weaknesses**
BL	Easy to construct (quick learning and low computational overhead); low sensitivity to missing data; recursive updating.	Low performance with clearly non-normal data or manifestly non homoscedastic distributions; poor calibration.
BQ	Easy to construct (quick learning and low computational overhead); low sensitivity to missing data; recursive updating.	Low performance with clearly non-normal data; poor calibration.
*k*NN	Very intuitive; no statistical assumption about the data; good classification if number of samples is large enough.	Critical choice of neighbourhood size and metric; large storage requirements and time consuming for large databases.
LR	Parsimony (few model parameters); interpretability of the parameters in terms of odds.	Outliers can affect results significantly; certain assumptions about predictors; difficult updating.
ISS	Very simple use in clinical practice; strong intuitive appeal; widespread use in heart surgery.	Worse performance than more complex models; difficult customization and updating.
ANN	No statistical assumption about data; ability to estimate non-linear relationships between input data and outputs.	Long training process; experience needed to determine network topology; poor interpretability; difficult updating.

BL, Bayes linear; BQ, Bayes quadratic; kNN, k-nearest neighbour; LR, logistic regression; ISS, integer scoring systems; ANN, artificial neural network.

1) Integer score models, application of which does not require a personal computer, are frequently preferred in clinical practice for their simplicity. However, this simplicity may undermine their predictive capacity which is generally worse than that of more complex models.

*2) k-*nearest neighbour algorithms do not make any statistical assumptions about data, are attractive from an interpretative point of view and are very easy to update with new data. Computational cost and the need for large data storage are weaknesses of this approach.

3) Logistic regression is a valid approach proven to give good predictive results in many clinical applications. As in most regression procedures, it is very sensitive to large linear correlations between the predictor variables in the model. LR models are not easily updated with new training data.

4) Artificial neural networks have intrinsic advantages with respect to common statistical models and the network architecture needed for clinical prediction problems is generally not too complex. However, the training process is problematical and updating by periodic retraining is inadvisable.

5) Bayesian models (especially Bayes quadratic models) seem a good compromise between complexity and predictive performance. Entering new correctly classified cases into the training set is a straightforward procedure, since it merely involves updating the mean vector and covariance matrix estimates using simple recursive relationships. A need for recalibration is generally a weakness of Bayes models.

Although it is essential to know model theoretical characteristics when a classification problem has to be solved by predictive models, the final choice of an appropriate model for a clinical scenario also calls for evaluation and comparison of the actual performance of several locally-developed competitive models using real experimental data in order to find satisfactory agreement between local needs and model response. In Part II of this study the above predictive models are applied and tested with real data of patients in a specialized cardiac postoperative ICU.

**Note: **This paper is accompanied by a Part II which gives a full account of an illustrative example [71].

## List of abbreviations

ANN = artificial neural network; AUC = area under the ROC curve; BL = Bayes linear; BQ = Bayes quadratic; CPDF = class-conditional density function; DS = direct score; ES = early stopping; FN = False negatives; FP = false positives; FPF = false-positive fraction; HL = Hosmer-Lemeshow; HS = Higgins score; ICU = intensive care unit; *k*NN = *k*-nearest neighbour; LOO = leave-one-out; LR = logistic regression; MSE = mean squared error; ROC = receiver operating characteristic; SE = sensitivity; SP = specificity; TN = true negatives; TP = true positives; TPF = true-positive fraction.

## Competing interests

The author(s) declare that they have no competing interests.

## Authors' contributions

All authors participated in the study plan and coordination. EB was concerned with epidemiology and biostatistical aspects of the study. GC and PB were concerned with medical informatics and biostatistical aspects of the study. SS, BB and PG were involved in clinical aspects. All authors read and approved the final manuscript.

## Pre-publication history

The pre-publication history for this paper can be accessed here:


